# Tumor microenvironment antigens

**DOI:** 10.1007/s00281-022-00966-0

**Published:** 2022-09-29

**Authors:** Mads Hald Andersen

**Affiliations:** 1 National Center for Cancer Immune Therapy (CCIT-DK), Department of Oncology, Copenhagen University Hospital Herlev, Borgmester Ib Juuls Vej 25C, 5th floor, DK-2730 Herlev, Denmark; 2 Department of Immunology and Microbiology, University of Copenhagen, Copenhagen, Denmark

**Keywords:** Tumor microenvironment antigens, TMA, Anti-regulatory T cells, Anti-Tregs, IDO, PD-L1, Arginase, TGF-beta, Immune modulatory vaccine

## Abstract

The identification and characterization of tumor antigens are central objectives in developing anti-cancer immunotherapy. Traditionally, tumor-associated antigens (TAAs) are considered relatively restricted to tumor cells (i.e., overexpressed proteins in tumor cells), whereas tumor-specific antigens (TSAs) are considered unique to tumor cells. Recent studies have focused on identifying patient-specific neoantigens, which might be highly immunogenic because they are not expressed in normal tissues. The opposite strategy has emerged with the discovery of anti-regulatory T cells (anti-Tregs) that recognize and attack many cell types in the tumor microenvironment, such as regulatory immune cells, in addition to tumor cells. The term proposed in this review is “tumor microenvironment antigens” (TMAs) to describe the antigens that draw this attack. As therapeutic targets, TMAs offer several advantages that differentiate them from more traditional tumor antigens. Targeting TMAs leads not only to a direct attack on tumor cells but also to modulation of the tumor microenvironment, rendering it immunocompetent and tumor-hostile. Of note, in contrast to TAAs and TSAs, TMAs also are expressed in non-transformed cells with consistent human leukocyte antigen (HLA) expression. Inflammation often induces HLA expression in malignant cells, so that targeting TMAs could additionally affect tumors with no or very low levels of surface HLA expression. This review defines the characteristics, differences, and advantages of TMAs compared with traditional tumor antigens and discusses the use of these antigens in immune modulatory vaccines as an attractive approach to immunotherapy. Different TMAs are expressed by different cells and could be combined in anti-cancer immunotherapies to attack tumor cells directly and modulate local immune cells to create a tumor-hostile microenvironment and inhibit tumor angiogenesis. Immune modulatory vaccines offer an approach for combinatorial therapy with additional immunotherapy including checkpoint blockade, cellular therapy, or traditional cancer vaccines. These combinations would increase the number of patients who can benefit from such therapeutic measures, which all have optimal efficiency in inflamed tumors.

## Traditional tumor-associated antigens and tumor-specific antigens

T cells can recognize tumor-associated antigens (TAAs), peptides derived from intracellular proteins expressed on the tumor cell surface as part of the major histocompatibility complex (MHC). Most antigens have been identified through the spontaneous anti-tumor immune response associated with cancer. Tumor antigens can be derived from viruses or from abnormally expressed or mutated proteins and are typically divided into five categories (reviewed in [1]), described below.

(i) *Viral antigens*: Several types of cancers are related to viral infections, including with human papillomavirus (HPV). In cervical cancer, HPV E6 and E7 proteins are produced inside tumor cells and give rise to antigenic surface peptides that T cells can detect. Therapeutic cancer vaccinations for HPV-related cancers have focused especially on long HPV peptides, which can elicit an increase in the number and activity of HPV-16-specific CD4 and CD8 T cells [2, 3].
(ii) *Mutated neoantigens*: The immune system can recognize antigens derived from mutated proteins, categorized as shared antigens that are common to several tumor types, or as patient-specific antigens. Shared antigens include proteins encoded by genes that often are mutated in cancer, including BRAF, P53, KRAS, NRAS, and Jak2 [4–8]. In addition, antigens can arise because of frame-shift mutations, such as CalR [9, 10], or chromosomal translocations, such as BCR-ABL or ETV6-AML1 [11, 12]. Patient-specific antigens comprise the products of the numerous mutations revealed by exome sequencing of tumors [13, 14]. Mutated antigens can derive from alterations in oncogenes or tumor suppressor genes (driver mutations) or from mutations in various genes that are not directly associated with tumor suppressor genes or oncogenes (carry-on mutations).
(iii) *Cancer-testis antigens*: Cancer-testis antigens (reviewed in [15]) include MAGE, BAGE, and GAGE [16–18]. These antigens are expressed in a wide variety of cancer types but not in any normal tissues except testis germline cells. Testis cells do not express class I human leukocyte antigen (HLA) molecules on their surfaces, so that until recently, cancer-testis antigens were considered to be “tumor specific” in the sense that T cells recognize only antigens presented by HLA. However, low levels of MAGE-A12 expression have been detected in brain cells, and neurological toxicity can be induced by anti-MAGE-A3/A9/A12 T cell receptor gene therapy, calling into question the tumor specificity of these antigens [19].
(iv) *Differentiation antigens*: Differentiation antigens are derived from proteins expressed in both cancer cells and the corresponding healthy tissue (e.g., melanoma cells and melanocytes). Most differentiation antigens have been identified in melanoma cells (e.g., Melan-A/MART-1, gp100/pmel17, tyrosinase, or TRP2 [20–22]) and prostate cancer cells (e.g., prostate-specific antigen and prostatic acidic phosphatase [23, 24]).
(v) *Overexpressed antigens:* The immune system also can recognize proteins that are overexpressed in cancer cells compared with normal tissue. Some examples are antigens derived from inhibitors of apoptosis proteins [25], such as survivin [26, 27], livin [28], and the Bcl-2 family [29–31], in addition to hTERT [32], HER2/neu [33], and WT1 [34, 35]. Various tumor types broadly express many overexpressed antigens, which often play an important role in tumor cell survival or growth, posing attractive targets for immunotherapy.

Tumor-specific antigens (TSAs) include viral antigens, mutated antigens, and cancer-testis antigens—although the latter categorization is debated, as noted in the MAGE-A12 example. TAAs include differentiation-related and overexpressed antigens. The central feature of almost all forms of anti-cancer immunotherapies is reliance on the host immune system to produce T cells that recognize tumor antigens. Research that aims to identify and characterize tumor antigens has focused on those expressed in tumor cells and not in healthy cells. Thus, increasingly, TSAs are gaining the most attention in immunotherapy studies because T cells that recognize TSAs leave normal tissues completely unscathed. Furthermore, tolerance mechanisms are not expected to affect immune responses against TSAs.

In the last decade, however, another type of T cell antigens has been identified. These are expressed in both tumor and non-tumor cells in the tumor microenvironment (TME), opening the way to overcoming the therapeutic difficulty of immune evasion that occur because cancer cells hijacks the immune-regulatory mechanisms that keep immune responses in check. In this review, we designate these antigens as tumor microenvironment antigens (TMAs) and illustrate how TMAs as therapeutic targets offer several advantages that differentiate them from traditional tumor antigens.

## Tumor microenvironment antigens

### Anti-regulatory T cells

In patients with metastatic melanoma, remarkably promising clinical efficacy has been shown from first-line treatment with a vaccine based on peptides derived from indoleamine 2,3-dioxygenase (IDO) and programmed cell death ligand (PD-L) 1 in combination with anti-programmed cell death protein 1 (PD-1) antibodies. The objective response rate with this combination was 80%, along with a complete response rate of 43%, superior to any other combinatorial therapy in patients with previously untreated melanoma [36]. Of note, both IDO and PD-L1 are expressed not only by melanoma cells but also by different non-malignant, immune regulatory cell types in the TME.

**Fig. 1:**
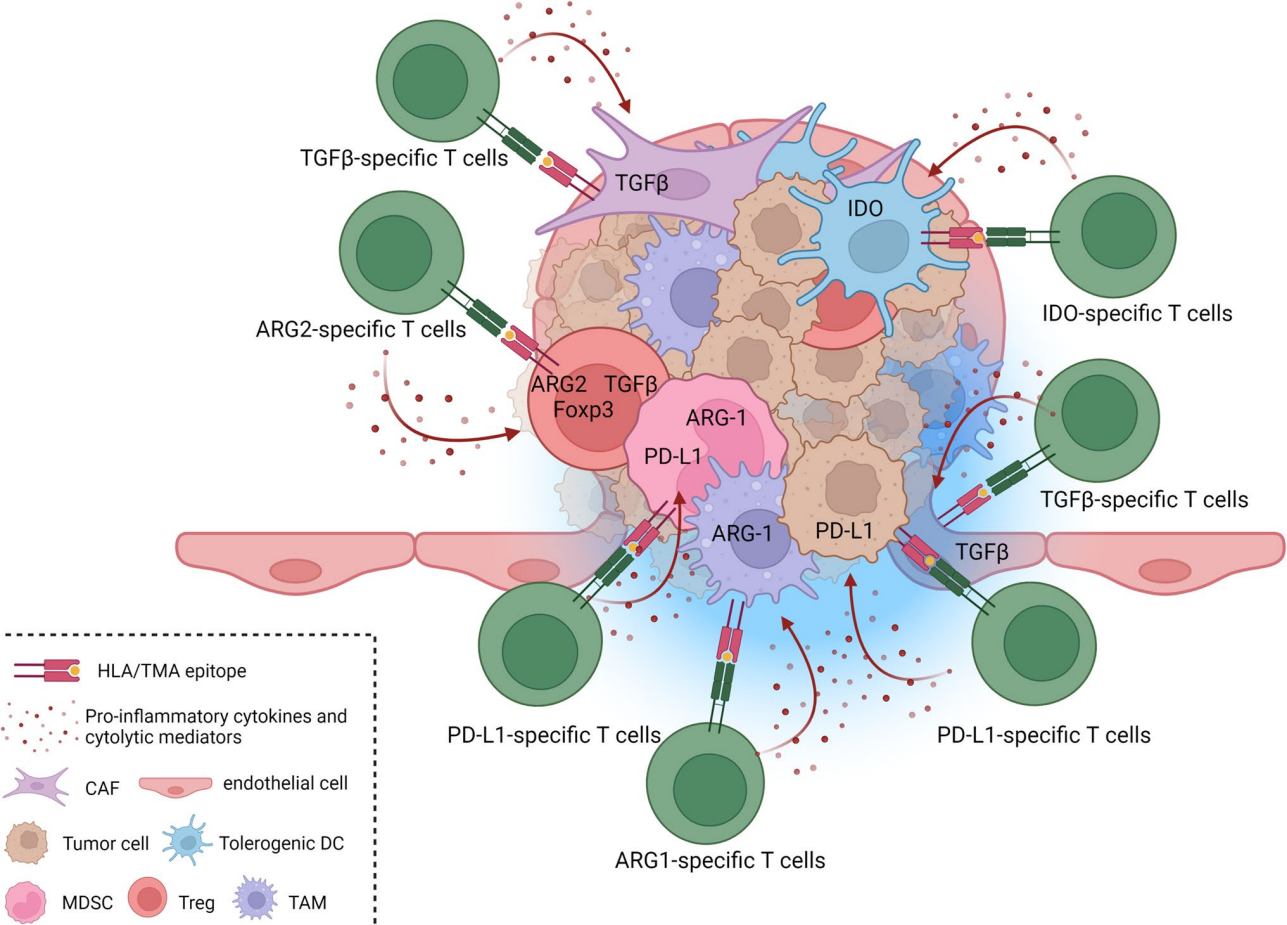
Anti-regulatory T cells (anti-Tregs) attacking TMA-derived epitopes expressed by different cells in the tumor microenvironment (TME). The TME comprises a mass of heterogeneous cell types, including myeloid cell populations such as myeloid-derived suppressor cells (MDSCs) and tumor-associated macrophages (TAMs) expressing TMAs (e.g., arginase-1 and PD-L1), as well as tumor-associated dendritic cells (DCs) expressing, e.g., IDO; regulatory T cells (Tregs) expressing, e.g., Foxp3, TGFβ, and arginase-2; cancer-associated fibroblasts (CAFs) expressing, e.g., TGFβ; endothelial cells expressing, e.g., vascular endothelial growth factor, survivin, and TGFβ; and malignant cells expressing, e.g., IDO, arginase, PD-L1, and TGFβ. The presence of the different cell types and the corresponding expression of TMAs within the TME varies with tumor origin and among individual patients. The importance of distinct TMA-specific T cells may also vary depending on tumor type, and immune modulatory vaccines should be designed accordingly. The Figure was created with BioRender.com.

In the last decade, studies have described self-reactive, pro-inflammatory T cells that specifically target such immune-suppressive cells and counteract a range of counter-regulatory feedback signals, particularly in patients with cancer [37]. The function of these T cells is to oppose regulatory cells, leading to their designation as anti-regulatory T cells (anti-Tregs) [38, 39]. Anti-Tregs thus are naturally occurring T cells that restrict the range of immunosuppressive signals mediated by regulatory cells. Anti-Tregs recognize HLA-restricted epitopes derived from proteins expressed by regulatory immune cells at inflammation sites, including IDO [40, 41] and tryptophan 2,3-dioxygenase (TDO) [42], PD-L1 and PD-L2 [43–47], arginase (ARG)1 and ARG2 [48–51], CCL22 [52], transforming growth factor (TGF)β [53, 54], and FoxP3 [55, 56] (Table 1). These proteins all play key roles in the regulation of immune responses as depicted below. The immune system works through a balance between stimulating and inhibitory mechanisms to protect the host by defeating the pathogen while preventing a harmful overreaction of the immune system. Unfortunately, this activation of the regulatory immune system also entails regulatory cells playing a crucial role in the pathogenesis of persistent infections as well as cancer. These antigens are thus commonly expressed across multiple immunosuppressive cell types, including tumor-associated macrophages (TAMs), myeloid derived suppressor cells (MDSCs), tolerogenic dendritic cells (DCs), regulatory T cells (Tregs), and cancer-associated fibroblasts (CAFs), in addition to cancer cells [57–60]. Thus, normal cells express these antigens under many inflammatory conditions [40, 61], as do different cells in the TME in the context of cancer [38]. This feature differentiates TMAs from traditional tumor antigens. As noted, TMAs can be derived from many types of proteins, and below, the different known types of TMAs are summarized. More types likely exist, and we recently suggested that galectins also may represent a novel type of TMA [62]. Figure 1 depicts a TME under attack from anti-Tregs recognizing different TMAs.

**Table 1:** Different categories of Tumor Microenvironment Antigens (TMAs)

Main TMA categories	Examples of TMAs	Main target cells in the tumor microenvironment
*1. Metabolic enzymes*	IDO	Tolerogenic DCs, MDSCs, tumor cells
	TDO	CAFs, epithelial cells, tumor cells
	ARG-1	TAMs, MDSCs, tumor cells
	ARG-2	Tregs, CAFs, tumor cells
*2. Checkpoint inhibitors*	PD-L1	TAMs, MDSCs, CAFs, tolerogenic DCs, tumor cells
	PD-L2	TAMs, CAFs, tumor cells
*3. Chemokines and cytokines*	TGFβ	CAFs, Tregs, TAMs, Tumor cells,
	CCL22	TAMs, tumor cells
*4. Transcription factors*	FoxP3	Tregs
*5. Traditional TAAs*	survivin	epithelial cells, tumor cells
	VEGF	epithelial cells, tumor cells

*Abbreviations*: *TMAs* tumor microenvironment antigens; *IDO* indoleamine 2,3-dioxygenase; *TDO* tryptophan-2,3-dioxygenase; *ARG1* arginase1; *ARG2* arginase2; *PD-L1* programmed cell death ligand 1; *PD-L2* programmed cell death ligand 2; *TGFβ* transforming growth factorβ; *VEGF* vascular endothelial growth factor; *DCs* dendritic cells; *MDSCs* myeloid derived suppressor cells; *TAMs* tumor-associated macrophages; *CAFs* cancer-associated fibroblasts; *Tregs* regulatory T cells

### TMA type 1: metabolic enzymes

In the TME, tumor metabolism often results in essential nutrient depletion in addition to accumulation of immune-suppressive metabolites [63–66]. L-Tryptophan is an essential amino acid required for protein synthesis, and tryptophan metabolites directly suppress immune reactions [67]. The enzymes IDO, IDO2, and TDO catalyze the degradation of L- and D-tryptophan, and T cells respond to low tryptophan levels via the serine/threonine-protein kinase GCN2, triggering proliferative arrest [68]. Likewise, expression of ARG1 and ARG2 enzymes in both cancer and immunosuppressive cells leads to the depletion of another important amino acid, L-arginine, from the TME, suppressing T cell–mediated anti-tumor immunity [65, 69]. Specific T cells react to these metabolic enzymes [40–42, 70–73].

IDO was the first metabolic enzyme to be described as a TMA, based on the finding that both CD4-and CD8-specific T cells react to IDO-derived HLA-restricted epitopes. We detected an association between patients harboring spontaneous CD4 and CD8 responses against IDO, indicating that class I– and class II–restricted IDO responses co-develop [40, 70–73]. IDO-reactive CD8+ T cells have been described as peptide-specific, cytotoxic effector cells [40, 73]. IDO-specific T cells thus lyse IDO+ cancer cell lines of different origins, including melanoma cells and ex vivo–enriched leukemia cells, exemplifying the universal character of TMAs shared among a variety of human cancers. IDO-reacting CD4+ T cells also respond specifically to DCs pulsed with IDO+ tumor lysates, emphasizing the cancer relevance of IDO-specific T cells [70]. IDO-specific CD4+ T cells have been described further as pro-inflammatory cells that release interferon (IFN)γ as well as tumor necrosis factor (TNF)α. Even more distinctive was our finding that IDO-specific T cells recognize and kill IDO+ immune cells [40]. These results demonstrate that IDO-specific T cells can indeed react to non-malignant cells, suggesting that these T cells have a potential immune modulatory role.

Indeed, we found that in reacting to IDO+ cells, IDO-specific T cells enhance other T cell responses [40]. For example, co-activation of IDO-specific, cytotoxic T cells boosted T cell immunity towards both viral and tumor-associated antigens. Similar effects was described in an animal model of cancer, in which IDO-based vaccination significantly enhanced immune responses against other tumor antigen–specific vaccines by downregulating IDO-expressing cell numbers in the TME [74].

IDO produces kynurenine, which may effectively hamper the immune response by binding the aryl hydrocarbon receptor, which favors the local formation of Tregs. Hence, targeting IDO-positive cells should indirectly decrease the number of Tregs. Indeed, the frequency of Tregs has been found to decrease when IDO-specific T cells are activated in human in vitro systems and in murine models [40, 74]. Because Tregs do not normally express IDO [67, 75, 76], this observation illustrates the principle that activating TMA-specific T cells also can affect non-target cells. Another study showed that vaccination against IDO-derived epitopes in an animal model of cancer exerted a therapeutic effect that synergized with anti-PD-1. In that model, IDO was expressed in myeloid cells in the TME, and this cell population decreased because of the vaccination [77]. Hence, the effect of IDO-specific T cells on T-cell immunity can be mediated both directly and indirectly. Although T cell reactivity has been detected towards the tryptophan-depleting enzymes IDO-2 [41] and TDO [42], these targets are much less studied than IDO-specific T cells.

As noted, the metabolic enzymes ARG1 and ARG2 are expressed in the TME of many tumors, leading to arginine depletion from the TME [66, 78–82]. L-Arginine deprivation downregulates expression of the T cell receptor ζ chain and decreases T cell cytokine production and proliferation. ARG1 is expressed especially in immunosuppressive myeloid cells including TAMs, MDSCs, and different DC subtypes [83]. Additionally, ARG2 is reported to be preferentially expressed by functional immunosuppressive Tregs in humans [84, 85]. These cells play a major role in the development of a suppressive microenvironment because they prevent effector lymphocyte proliferation at the tumor site [65]. We have described the existence of ARG1-specific T cells and demonstrated that they can recognize and react against DCs in addition to B cells expressing ARG1 [48, 50, 86]. Recently, we showed that these pre-existing T-cell responses against ARG1 are part of the T cell memory repertoire [49]. Likewise, naturally occurring effector T cells specific to ARG2 have been described [51]. We found that cytotoxic ARG2-specific CD8+ T cells can specifically recognize ARG2-expressing activated Tregs along with ARG2-expressing cancer cell lines [85], highlighting the anti-regulatory function of these effector T cells. Furthermore, ARG1- or ARG2-based vaccination in several murine models of cancer can activate specific T cells and induce tumor growth control [51, 87]. ARG-targeting therapeutic vaccines change the cells of the TME with a resulting increased infiltration of T cells and a shift in the M1/M2 ratio of tumor-infiltrating macrophages [87]. In addition, we have observed both decreased ARG1 expression and reduced suppressive function of tumor-educated myeloid cells after vaccination. Of note, ARG-targeting vaccines function in synergy with anti-PD-1 [87]. Taken together, these results put forward ARG-based immune modulatory vaccines as a novel therapeutic modality against cancer.

### TMA type 2: checkpoint inhibitors

The importance of PD-1/PD-L1 pathway regulation in T cell immunity has been highlighted by the tremendous success of blocking this pathway in cancer [88]. PD-1 is an inhibitory receptor, and signaling through PD-1 renders T cells functionally non-reactive against its cognate target antigen [89]. PD-1 expression by tumor-infiltrating T cells is a major inhibitor of the spontaneous anti-tumor immune response in patients with cancer [90]. PD-1 and its ligands PD-L1 and PD-L2 play central roles in the development of an immune-inhibitory TME that protects malignant cells from immune cell–mediated death [91]. Both PD-L1 and PD-L2 can be described as TMAs because they are recognized by specific T cells in patients with cancer [44, 45, 47, 92, 93]. As for the actual checkpoint proteins, the main focus has been on PD-L1-specific T-cell recognition, and PD-L2 has been less studied. Natural PD-L1-reactive T cells can be readily detected in the peripheral blood of patients with cancer [44, 45]. We additionally found that PD-L1-specific T cells kill PD-L1-expressing melanoma cells and cutaneous T-cell lymphoma cells [44, 46]. In agreement with these findings, Minami et al. described the lysis of PD-L1+ HLA-A24+ renal carcinoma cells by HLA-A24–restricted PD-L1-specific T cells [94]. Additionally, PD-L1-specific T cells can recognize non-malignant immune cells in a PD-L1 concentration-dependent manner, highlighting the potential immune modulatory role of these T cells [44]. PD-L1 is expressed in high amounts mainly in immune-suppressive cells, but it can be expressed by antigen-presenting cells, placental cells, non-hematopoietic cells, and even activated T cells in an inflammatory microenvironment, as both type I and II IFNs induce PD-L1 expression [95–100].

To investigate the immune modulatory functions of PD-L1-specific T cells, we added them to cultured peripheral blood mononuclear cells that had been stimulated a week earlier with known immune-dominant viral epitopes from, e.g., influenza and Epstein–Barr virus. The result was an immense increase in the number of virus-specific CD8+ T cells [43], an effect confirmed in other co-stimulation assays. For example, we observed a significant increase in the numbers of virus-specific T cells in cultures co-stimulated with the PD-L1 peptide epitope compared with cultures co-stimulated with an irrelevant HIV epitope [93]. Likewise, co-stimulation with a PD-L1 epitope resulted in increased immune reactivity towards a cellular-based cancer vaccine [101]. These results suggested that PD-L1-specific T cells may assist with the effector phase of an immune response by providing pro-inflammatory cytokines at the site of inflammation in addition to directly removing PD-L1-expressing regulatory immune cells that inhibit PD-1+ effector T cells.

The primary role of the PD-1 pathway is believed to be regulation of effector T-cell responses to control tissue damage. Thus, this protective pathway is more important after activation rather than at the initial T-cell activation stage [89, 102]. Accordingly, the presence of PD-L1-specific T cells during the activation phase of an immune response may not have a supportive function for a pro-inflammatory response, as seen in the effector phase. In fact, we found that stimulation with viral epitopes in the presence of already activated PD-L1-specific T cells resulted in decreased numbers of viral-specific T cells after 2 weeks of culture [43], possibly because of the expression of PD-L1 on potent antigen-presenting cells. PD-L1 also can be expressed on activated T cells, however we note that PD-L1+ T cells mainly exert tolerogenic effects on tumor immunity and show tumor-promoting properties, suggesting that targeting this immune population would indeed be beneficial [95]. The effects of PD-L1-specific T cells thus might vary depending on the expression of both PD-1 and PD-L1 and their effects on the microenvironment and the state of the immune response. These factors should be considered when targeting PD-L1 as a TMA.

### TMA type 3: chemokines and cytokines

The cytokine TGFβ is a key immune regulatory molecule [59] that we recently identified as a novel target for anti-Tregs, as we characterized T cells that can directly recognize TGFβ-expressing cells [53, 54]. Under normal conditions, TGFβ regulates T-cell immunity and DC function, induces tolerance, and controls the extent of inflammation. In the context of cancer, various regulatory cells such as Tregs, TAMs, and CAFs accumulate in the TME and produce high levels of TGFβ [59]. Cancer cells frequently develop non-response to the cytostatic effects of TGFβ and selectively exploit its role as a promoter of vascularization, tissue invasion, and metastasis. In contrast, tumor-combating immune cells such as cytotoxic T cells, tumor-associated neutrophils, and natural killer cells are susceptible to the suppressive effects of TGFβ, which greatly impairs their activation, recruitment, and functionality [59]. Furthermore, TGFβ expression drives decreased efficiency of immune checkpoint inhibitors in many patients because TGFβ triggers immune exclusion in various cancers [103].

We investigated whether our characterization of TGFβ-specific T cells could be used for a TGFβ-based peptide vaccination strategy. In our *in vivo* preclinical studies in a murine “cold” tumor model of pancreatic cancer, we showed that TGFβ-derived peptide vaccination controlled tumor growth [104], targeting immunosuppression in the TME by polarizing its cellular composition towards a more pro-inflammatory phenotype. Our findings support the feasibility and potential of TGFβ-derived peptide vaccination as a novel immunotherapeutic approach and highlight TGFβ as a highly attractive TMA for anti-cancer therapy.

Another important immunosuppressive cytokine, CCL22, is a macrophage-derived immunosuppressive chemokine that recruits mainly Tregs to the TME through the CCL22:CCR4 axis [105]. CCL22 thus suppresses anti-cancer immune responses in cancer of different origins [106, 107]. It also is a TMA that can be recognized by specific T cells [52]. CCL22-specific T cells can recognize and kill CCL22-expressing breast and colon cancer cells, as well as lysed acute myeloid leukemia cells in a CCL22 concentration–dependent manner. *In vitro* experiments have shown that CCL22-specific T cells can affect the TME by decreasing CCL22 levels [52]. In other work, vaccination with CCL22-derived peptides in *in vivo* mouse models of cancer induced CCL22-specific T-cell responses [108] that slowed tumor growth and extended survival. CCL22-based vaccination further modified the TME by changing the cellular composition of immune cells that infiltrated the TME, including increasing the CD8:Treg ratio [108]. These findings suggest that a TMA vaccine based on CCL22 may directly target cancer cells and TAMs, which should decrease Treg recruitment into the TME and enhance anti-cancer immunity.

### TMA type 4: transcription factors

Foxp3 expression is the classical marker of Tregs [109], and the protein may function as a TMA, especially in murine models. In an animal model of cancer, Gilboa and colleagues first described FoxP3-based vaccination induction of FoxP3-specific T cells that eliminated FoxP3+ Tregs while enhancing anti-tumor immunity [110]. A similar study in an atherosclerosis model likewise showed that FoxP3-specific T-cell responses substantially decreased the number of FoxP3+ Tregs, resulting in increased atherosclerotic lesion formation [111]. The correlation of FoxP3 and Tregs, however, is much more complex in humans compared with mice, as activated conventional T cells also express FoxP3 [112]. Nevertheless, we found that humans show natural CD8 reactivity towards FoxP3 [55, 56]. FoxP3-specific anti-Tregs recognize Tregs and kill malignant T cells expressing high FoxP3 levels, suggesting that vaccination against FoxP3 could be useful in patients with lymphoma involving FoxP3+ malignant T cells. The pro and cons of a FoxP3-based vaccine in humans remain unclear because of the potential for side or unwanted effects.

### TMA type 5: traditional TAAs

Some traditional TAAs also are expressed on non-tumor cells in the TME, and targeting these antigens might likewise lead to a broader attack in the TME. For this reason, one can argue that some TAAs could also be considered TMAs. One study has shown that different inflammatory conditions induce abnormal expression of some TAAs in non-malignant epithelial cells [113], promoting spontaneous immunity to these TAAs in healthy individuals with no history of cancer. In the same work, TAAs such as carcinoembryonic antigen, HER2/neu, and MUC1 were upregulated in epithelial cells in response to pro-inflammatory cytokines, and the TAAs seppin B1 and SOD2 were overexpressed in pre-malignant and malignant breast tissues, and in the context of inflammatory conditions in the colon, stomach, and liver. Additionally, targeting TAAs expressed on vascular epithelial cells leads to inhibition of angiogenesis in the tumor [114, 115]. Several clinical trials have used vascular endothelial growth factor (VEGF) as a target in angiogenesis [116–118], but other similar TAAs have been described. For example, a DNA vaccine that targets the universally expressed TAA survivin induces angiogenesis suppression in lung tumor eradication [119].

## Therapeutic targeting of TMAs

### Class I and II TMAs

Many recent anti-cancer vaccination strategies based on TSAs (or TAAs) have focused primarily on generating CD8 T-cell responses because these cells kill cancer cells [120]. In contrast, TMA-based vaccinations should focus on both CD8 and CD4 responses. The major aim of a TMA-based vaccination is to modulate the immune repertoire and convert an immunosuppressive environment into a pro-inflammatory environment. In a therapeutic setting, the release of pro-inflammatory cytokines from TMA-specific CD4 T cells may be as important as the TMA-specific CD8-mediated killing of target cells. In a study already described above, patients with melanoma received long peptides from IDO and PD-L1 that contained both CD8 and CD4 epitopes in combination with anti-PD-1 therapy. The results showed that the induction of a pro-inflammatory TME was correlated with the re-polarization of innate immune cells, measured as an increase in class II HLA expression [36].

Activation of HLA class I–restricted cytotoxic CD8 T cells can lead to the direct targeting and elimination of cells that express the target antigens. Indeed, *in vitro* studies have shown that TMA-specific CD8 T cells can lyse many different cell types, including melanoma and myeloid cells [40, 42–46, 52, 70, 73, 93]. Furthermore, in animal models of cancer, vaccinations with IDO epitopes have shown therapeutic effects correlated with reductions in IDO-expressing myeloid cells in the TME of the CT26 colon cancer model [76]. Likewise, in a pancreatic cancer model, TGFβ vaccination decreased the TGFβ protein level in the TME [104].

TMA-specific CD4 T cells are potent expressors of immune-stimulatory cytokines. Consequently, these cells can locally reprogram the TME to favor tumor rejection by supporting anti-tumor T-cell responses and stimulating antigen presentation. Many immune regulatory cells can be reverted by changing the environment; for example, M2 (TAMs) can be reverted to M1 macrophages [121]. Upon encountering TMA-expressing target cells, CD4 T cells can secrete the pro-inflammatory cytokines IFNγ and TNFα [45, 49–51, 70]. IFNγ and TNFα can stimulate or activate other anti-tumor immune responses (both adaptive and innate) and can promote antigen presentation, which supports tumor recognition and elimination. The importance of combining MHC I– and MHC II–restricted T-cell epitopes in TMA-based immune modulatory vaccines has been shown in animal models of cancer in both the IDO-based and TGFβ-based vaccine settings [77, 104].

### Targeting of HLA- and TMA-negative tumor cells

Many tumor cells downregulate surface HLA expression to escape immune system surveillance. However, HLA often can be reintroduced in a pro-inflammatory microenvironment. Thus, immunological therapies that target non-transformed cells with consistent HLA expression might activate pro-inflammatory cells in the TME, in turn re-inducing HLA expression on tumor cells. Additionally, in inflammatory settings, TMAs such as PD-L1 and IDO are upregulated as a counter response to dampen the local immune response. In the context of a TMA-based vaccination, however, these upregulated suppressive molecules serve as targets for the TMA-specific T cells, which could lead to additional T-cell–promoted inflammation and sustain an overall pro-inflammatory shift in the microenvironment. Indeed, pre-incubating target cells with IFNγ increases their susceptibility to recognition by both IDO- or PD-L1-specific T cells [40, 44]. This potential also should be considered in the treatment of non-inflamed tumors in which TAMs, MDSCs, or CAFs might express high amounts of other TMAs, such as ARG1 or TGFβ. If an ARG1- or TGFβ-based vaccine can activate ARG1- or TGFβ-specific T cells, pro-inflammatory cytokines produced during such an immune attack against immune-suppressive cells could make the cells susceptible to further T-cell attack by IDO- and PD-L1-specific T cells. Thus, combining different TMAs in vaccine cocktails is a highly attractive approach that might yield synergistic effects [122]. The pro-inflammatory activity of immune modulatory vaccines also would be relevant in combination with immune checkpoint blockade immunotherapy. Combinatorial therapy with immune modulatory vaccines and checkpoint blockade would be expected to increase the number of patients who can benefit from checkpoint blockade [36], a strategy with best efficiency in inflamed (or “hot”) tumors [88].

In contrast to strategies targeting TSAs or TAAs, TMA-based vaccinations may have therapeutic effects, regardless of whether the tumor cells themselves express the cognate TMA targets. For example, Dey et al. reported that IDO-based vaccination showed a therapeutic effect in the CT26 cancer model. They demonstrated that CT26 tumor cells did not express IDO, but that myeloid cells in the TME did so, and this cell population decreased as a result of the vaccination [76]. Similarly, in a small clinical trial in patients with basal cell carcinoma (NCT03714529), a PD-L1 peptide-based vaccination induced regression in tumors, even though PD-L1 was expressed only by immune cells in the TME and not by tumor cells [123].

### Safety concerns in connection with TMA-based vaccination

TMAs are self-proteins and therefore expressed in many cell types. As discussed above, both PD-L1 and IDO are even induced by IFNs as a counter-response to inflammation. Regarding safety issues, this property provides a mechanism that ensures immune homeostasis, which keeps IDO/PD-L1-specific T cells in check. We have shown that the cytotoxicity of circulating IDO-specific T cells towards IDO-expressing malignant or immune cells was similar between IDO-specific T cells isolated from healthy individuals and those from patients with cancer [40]. Furthermore, we have described a direct link between inflammation and expansion of these cell populations. Th1-mediated inflammation signals, such as IFNγ, spontaneously lead to the expansion of IDO- and PD-L1-specific immune cells [40, 61]. IDO/PD-L1-specific T cells therefore expand as part of the response to inflammation and can function as helper cells at the inflammation site, where they also can aid in the response to infected cells. The expansion of IDO/PD-L1-specific T cells in response to inflammatory measures and the counter regulatory expression of IDO and PD-L1 illustrates that these T cells are tightly regulated and not inducing toxicity in vaccinated patients. The risk of triggering autoimmune-related adverse events by vaccination against these TMAs thus appears to be minimal, as was confirmed in the first clinical trial of IDO vaccination in patients with non-small cell lung cancer [124]. The median overall survival was >2 years, higher than the 8 months that were observed in a similar control cohort. Three of the fifteen patients were still alive at 6 years, for an overall survival of 20% at that follow-up. One of these three patients was excluded because of progression after 11 months, but the remaining two continued vaccinations every 4 weeks for 5 years, each receiving 56 vaccines in total. The vaccine was well tolerated for all 5 years, and the presence of IDO-specific T cells was detected during treatment [125].

Similarly, we conducted a phase I PD-L1 vaccination first-in-human study including 10 patients with multiple myeloma. The patients were vaccinated with a PD-L1-derived peptide as a consolidating treatment after standard high-dose chemotherapy, allowing 15 vaccinations over the course of a year [126]. All adverse reactions to the PD-L1 vaccine were below common toxicity criteria grade 3, and most were grade 1-2 injection site reactions. The total rate of adverse events was as expected for the population. All patients exhibited PD-L1-specific immune responses [126]. Even when combined with anti-PD-1 therapy, the systemic toxicity profile of IDO- and PD-L1-based vaccination was comparable to that of anti-PD-1 monotherapy [36]. However, humoral recognition of PD-L1 has been described in rheumatoid arthritis [127], suggesting that uncontrolled B cell immunity towards PD-L1 may be involved in autoimmunity.

Other TMAs such as ARG1 and TGFβ are not induced by inflammation. T cells specific for such TMAs function, however, only in very immunocompromised microenvironments, which may explain why ARG1- or TGFβ-targeting therapeutic vaccines can activate specific immunity in animal models of cancer without causing associated side effects or systemic toxicity [87, 104]. These T cells also exist in the periphery without introducing toxicity even in healthy individuals. Thus, TMA-specific T cells, such as CD8+ and CD4+ T cells specific to ARG1, ARG2, TGFβ, IDO, and PD-L1, can all be found in peripheral blood lymphocytes of healthy donors [44, 45, 51, 53, 73, 128]. Naturally occurring T cells (anti-Tregs) that recognize TMAs therefore must function as a normal part of the immune system, killing immunosuppressive cells to dampen local immune suppression. Anti-Treg levels are kept in a delicate balance with regulatory immune cells to maintain immune homeostasis [37].

## Conclusions

The targeting of TMAs offers a different therapeutic approach from targeting TSAs. TMA-specific T cells might directly kill not only tumor cells but also other regulatory cells. In addition, they might reprogram regulatory cell populations by releasing pro-inflammatory cytokines into an immunosuppressive microenvironment. TMAs could be further classified into different subcategories based on which type of normal cells express the antigen at sufficient levels to mediate an immune attack. Moreover, different TMAs could be combined in anti-cancer immunotherapies to attack tumor cells directly and modulate local immune cells to create a tumor-hostile microenvironment and inhibit tumor angiogenesis. Immune modulatory vaccines offer an attractive approach for combinatorial therapy with additional immunotherapy including checkpoint blockade, cellular therapy, or traditional cancer vaccines. These approaches likely would increase the number of patients who can benefit from such therapeutic measures, which all have optimal efficiency in inflamed tumors.
